# Supplementary material for: Associations between attention‐deficit hyperactivity disorder (ADHD) symptom remission and white matter microstructure: A longitudinal analysis
Source: JCPP Adv. 2021 Nov 2;1(3):e12040. doi: 10.1002/jcv2.12040 (PMC9012480; doi:10.1002/jcv2.12040)
Supplement: Supplementary file 1 — Supporting Information S1docx [file JCV2-1-e12040-s001.docx]

# Supporting Information

| **Model**  **1** | FA_TP1_ | ~ | ΔCPRS | + | age_TP1_ | + | Δage | + | sex | + | CPRS_TP1_ | + | head motion_TP1_ |  |  |
| --- | --- | --- | --- | --- | --- | --- | --- | --- | --- | --- | --- | --- | --- | --- | --- |
| **Model**  **2** | FA_TP2_ | ~ | ΔCPRS | + | age_TP1_ | + | Δage | + | sex | + | CPRS_TP1_ | + | head motion_TP2_ |  |  |
| **Model**  **3** | ΔFA | ~ | ΔCPRS | + | age_TP1_ | + | Δage | + | sex | + | CPRS_TP1_ | + | head motion_TP1_ | + | head motion_TP2_ |

### **Table S1.** Composition of our three general linear models. We essentially have a cross-lagged design with fractional anisotropy (FA) as the dependent variable. The difference in CPRS (ΔCPRS=CPRS_TP1_–CPRS_TP2_) is the predictor variable in all models. For each model, CPRS score could be the inattention, hyperactivity-impulsivity, or combined score. The outcome variables of these models are, respectively: FA at baseline (TP1), FA at follow-up (TP2), and change in FA. Permutation analysis (PALM) necessitated that we kept the (TBSS output) FA image as the dependent variable. We covaried for participant baseline age, change in years of age, sex, baseline CPRS symptom score, and framewise displacement head motion at the relevant FA time-point in the dependent variable.

|  |  |  |  | **MNI (Peak voxel)** | | |  |  |
| --- | --- | --- | --- | --- | --- | --- | --- | --- |
| **Exploratory model** | | **WM**  **tract** | **N_voxels_** | **X_COG_** | **Y_COG_** | **Z_COG_** | ***t_max_*** | ***P_FWE_*** |
|  |  |  |  |  |  |  |  |  |
| **∆FA ~ ∆CPRS_HI_** | | lIFOF | 508 | -29 | 24 | 17 | 0.441 | 0.041 |
|  |  | lIFOF | 376 | -17 | 31 | -10 | 0.613 | 0.035 |
|  |  | Fmin | 339 | -18 | 50 | 0 | 0.585 | 0.042 |
|  |  | lUNC | 174 | -25 | 17 | -8 | 0.564 | 0.045 |
|  |  | lCST | 158 | -22 | -13 | 8 | 0.562 | 0.047 |
|  |  | Fmin | 22 | -20 | 38 | 21 | 0.563 | 0.049 |
|  |  | CCG | 17 | -17 | 32 | 23 | 0.562 | 0.049 |
|  |  | lIFOF | 11 | -28 | 15 | -1 | 0.563 | 0.049 |
|  |  | Fmin | 11 | -18 | 46 | 17 | 0.566 | 0.049 |
|  |  | Fmin | 9 | -12 | 41 | -17 | 0.563 | 0.049 |
|  |  |  |  |  |  |  |  |  |

### **Table S2.** Exploratory *post-hoc* analysis results. Greater HI symptom decrease was associated with a larger decrease in FA over time in several WM clusters. P-values reported here were not adjusted for multiple testing. lIFOF: left inferior fronto-occipital fasciculus, Fmin: forceps minor of the corpus callosum, lUNC: left uncinate fasciculus, CCG: cingulum cingulate gyrus.

|  |  |  | **MNI (Peak voxel)** | | |  |  |  |
| --- | --- | --- | --- | --- | --- | --- | --- | --- |
| **Model** | **WM tract** | **N_voxels_** | **X_COG_** | **Y_COG_** | **Z_COG_** | ***t_max_*** | ***P_FWE_*** | **age_TP1_ interaction *P*** |
|  |  |  |  |  |  |  |  |  |
| **FA_TP2_ ~ ∆CPRS_combined_** | lCST | 723 | -21 | -27 | 44 | 0.755 | 0.044 | 0.463 |
|  | lSLF | 579 | -33 | -20 | 38 | 0.881 | 0.038 | 0.808 |
| **FA_TP2_ ~ ∆CPRS_HI_** | lCST | 17 | -18 | -25 | 52 | 0.981 | 0.049 | 0.154 |
| **∆FA ~ ∆CPRS_HI_** | lIFOF | 508 | -29 | 24 | 17 | 0.441 | 0.041 | 0.667 |
|  | lIFOF | 376 | -17 | 31 | -10 | 0.613 | 0.035 | 0.055 |
|  | Fmin | 339 | -18 | 50 | 0 | 0.585 | 0.042 | 0.874 |
|  | lUNC | 174 | -25 | 17 | -8 | 0.564 | 0.045 | 0.131 |
|  | lCST | 158 | -22 | -13 | 8 | 0.562 | 0.047 | 0.130 |
|  | Fmin | 22 | -20 | 38 | 21 | 0.563 | 0.049 | 0.239 |
|  | CCG | 17 | -17 | 32 | 23 | 0.562 | 0.049 | 0.238 |
|  | lIFOF | 11 | -28 | 15 | -1 | 0.563 | 0.049 | 0.238 |
|  | Fmin | 11 | -18 | 46 | 17 | 0.566 | 0.049 | 0.239 |
|  | Fmin | 9 | -12 | 41 | -17 | 0.563 | 0.049 | 0.237 |

### **Table S3.** Interaction effects of ΔCPRS and age at TP1 for significant models.

| **ΔCPRS score** | **Spearman’s ρ** | ***P*** |
| --- | --- | --- |
| Combined | 0.038 | 0.709 |
| Hyperactivity-impulsivity | 0.098 | 0.331 |
| Inattention | -0.007 | 0.948 |

### **Table S4.** No significant correlation (Spearman’s rho) between difference in CPRS score (ΔCPRS=CPRS_TP1_–CPRS_TP2_) and right-handedness in our sample.

### **Figure S1.** Processing pipeline. During pre-processing, DWI images were realigned and corrected for residual eddy current and for motion artefacts using robust tensor modelling (PATCH)(Zwiers, 2010). Diffusion tensor characteristics and FA values were calculated for each voxel(Behrens et al., 2003). After pre-processing, we used a custom longitudinal TBSS pipeline adapted from others to create non-biased individual subject templates (Madhyastha et al., 2014).


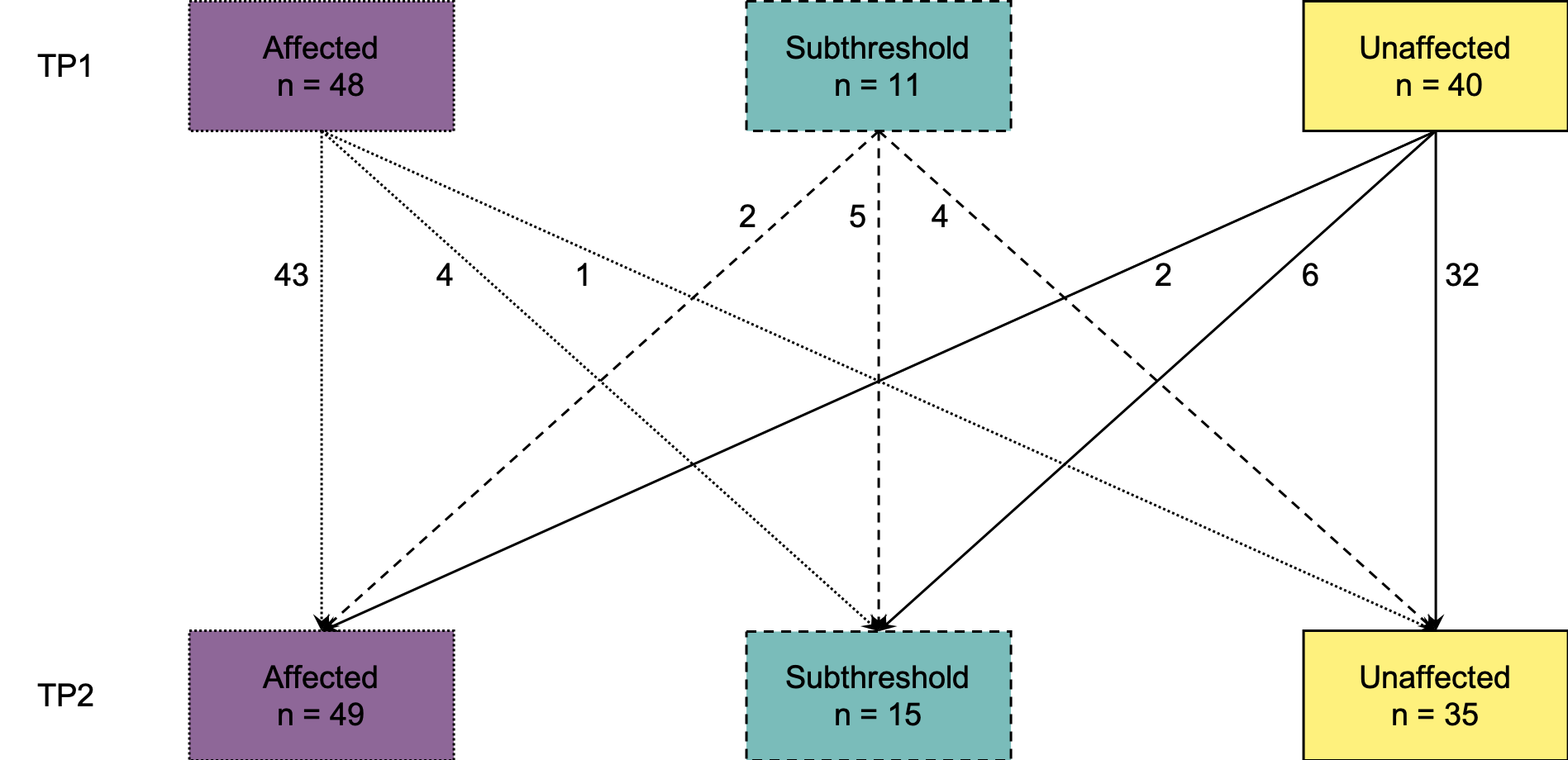


### **Figure S2.** Changes in the diagnostic make-up of our study sample (N=99). Although the total number of participants remained constant, the number of individuals in each diagnosis group changed between time-point 1 (TP1) and time-point 2 (TP2).


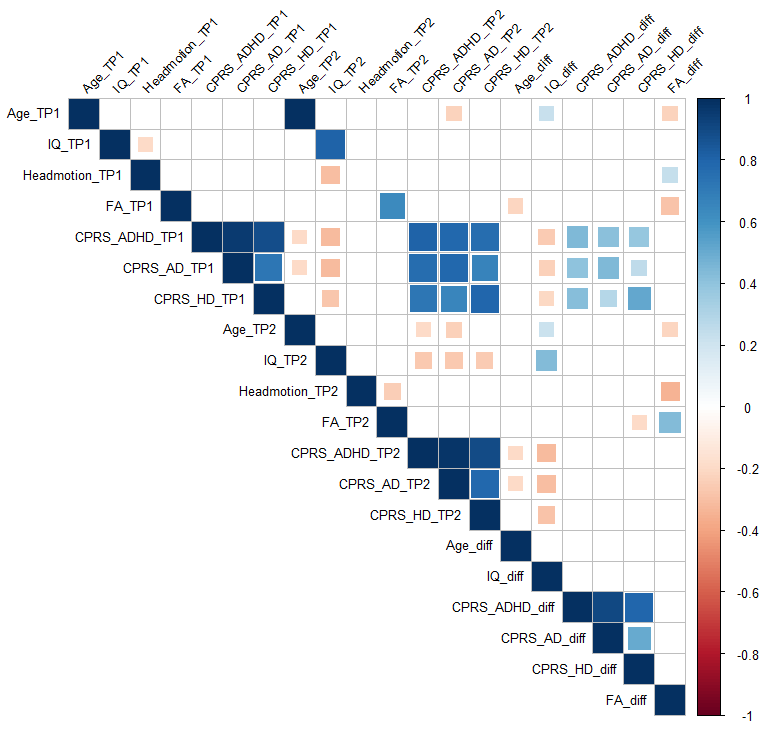


### **Figure S3.** Spearman correlation matrix of independent and dependent variables, as well as covariates. These correlation tests were performed before the main analyses. The color intensity of each box indicates the magnitude of the correlation. Positive correlations are presented in blue and negative correlations in red. The size of the box indicates its significance, with significant correlations filling each square completely.

### **Figure S4.** Line plot illustrating individual changes in average whole-brain fractional anisotropy (FA) from baseline to follow-up, grouped by never affected participants (blue) versus affected participants (including subthreshold). Here, affected individuals are further split along the median into two groups according to whether their change in combined Conners Parent Rating Scale score (ΔCPRS) was above (orange) or below (green) the affected sample’s median of that change.


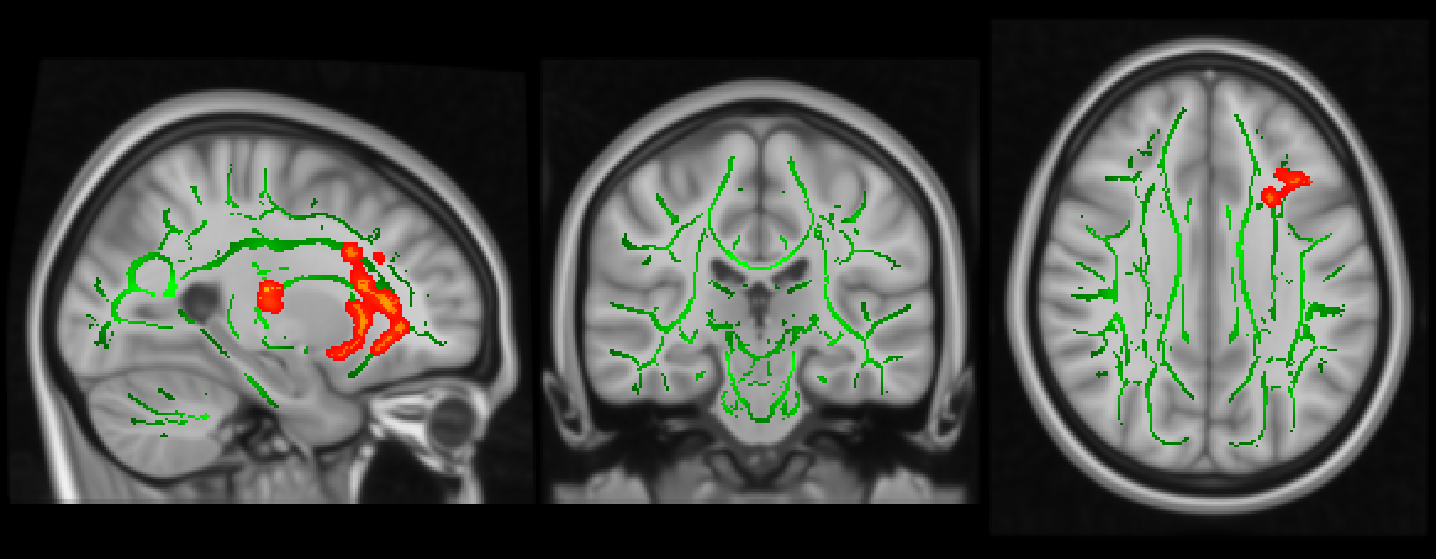


### **Figure S5.** Exploratory dimensional TBSS analyses showing significant associations (red-yellow) between FA values and the CPRS scores over time. The mean FA skeleton across all subjects (green) was overlain on the MNI template image for presentation (x=-25, y=-25, z=31). Results were thickened for visualization (FSL “tbss_fill”) and presented here in radiological convention from sagittal, coronal, and axial perspectives, respectively. A more negative change in FA (i.e. more isotropic diffusion) over time was associated with more HI symptom remission in ten clusters spread over six WM tracts. See Table S2 for cluster statistics and locations.
